# Developmental evaluation of the healthy futures of Texas’ puberty curriculum: *On My Way*

**DOI:** 10.3389/fpubh.2024.1441326

**Published:** 2024-09-25

**Authors:** Elizabeth Schormann, Anthony Betori, Leah C. Neubauer

**Affiliations:** ^1^Feinberg School of Medicine, Northwestern University, Evanston, IL, United States; ^2^Healthy Futures of Texas, Bloomberg Fellow at Johns Hopkins Univeristy, San Antonio, TX, United States; ^3^Division of Public Health Practice, Department of Preventative Medicine, Feinberg School of Medicine, Northwestern University, Evanston, IL, United States

**Keywords:** developmental evaluation, puberty education, adolescent, curriculum and instruction, pilot program

## Abstract

**Background:**

This paper describes a developmental evaluation (DE) of a pilot of a puberty curriculum that was implemented in grades four to six in San Antonio, Texas. The pilot evaluation assessed the initial feasibility and acceptability of curricular components. The DE framework guided the questions in an ever-changing environment where new tools were created as the situation called for them (10).

**Methods:**

The evaluation team utilized purposive sampling methods, surveys, and facilitator notes to guide the collaborative process. Both Google and Microsoft platforms were used for analysis and collection of findings.

**Results:**

Facilitator notes and surveys revealed that while comfortable leading sessions, there were still issues in timing and student comprehension. From a student’s point of view, while many (60%) reported feeling uncomfortable during lessons, a greater majority (80%) reported learning something from the sessions.

**Conclusion:**

DE was a crucial piece of the pilot sessions and revision process despite any limitations. A user-focused and adaptable evaluation generated greater opportunities for positive change within the curriculum and its delivery.

## Introduction

### Background: why puberty education?

Puberty is a temporary life stage that serves as a foreground for important changes within a young person’s emotional, physical, and social self; defined as “a developmental period in which hormonal changes cause rapid physical alterations in the body … the average age range for reaching puberty is between 9 and 14 for girls and between 10 and 17 years for boys” [(1): Physical Changes in Adolescents]. While it is a component of sex education, puberty education programs are nested in health and sexuality curriculums that are specific to an adolescent’s development during puberty; furthermore, puberty education can expand beyond a set curriculum into interventions that have a focus on behaviors and attitudes that align with this stage of development (2). Puberty is situated at the beginning of adolescence, marking the “transition from childhood to adulthood” (3). Recognizing this connection between life stages in curriculum-based educational programs has positive effects on an adolescent’s life, including reduced risk-taking and frequency of sexual intercourse, delayed initiation of sex, and increased use of contraceptives (4).

Adolescents should have access to this information since puberty typically occurs while in school, making it an opportune time to build a strong foundation as they grow and change (4). An inclusive, comprehensive sexual health education program allows youths to make informed decisions regarding their bodies and relationships confidently. It also reduces the number of adolescents who feel alone as they navigate through the rapid changes of puberty (5). Without a comprehensive puberty education, adolescents can be left feeling anxious. For example, menstruation is a neglected topic of discussion, which leaves stigma and general misconceptions to guide an adolescent as they begin this stage of maturation (4). However, with an inclusive and comprehensive program, adolescents can begin to build healthy habits based on facts rather than assumptions.

There is a lack of consensus on how puberty and sex education are to be taught, resulting in states developing different regulations for teaching this information in schools. Only 17 states that teach puberty and sex education require it to be medically accurate (6). In 2019, it was found that only 21% of public elementary schools and 45% of middle schools provide some level of puberty education, typically being grouped in the larger sphere of sex education. This number can dwindle further when opt-in/opt-out programs are accounted for (2).

The new Healthy Futures of Texas curriculum *On My Way* strives to be an inclusive and comprehensive program. It was important to make that decision since puberty education lacks evidence-based practices as few programs have been rigorously evaluated (2). As a result, universal goals of puberty education have yet to be firmly established, explaining why rigorous evaluation is needed. This is not to say that current puberty programs are not effective, but that there are limited formal evaluations of puberty education that are facilitated in the classroom (2).

### Background: what is developmental evaluation?

DE is “evaluation processes and activities that support program, project, product, personnel, and/or organizational development,” with the evaluator as an integrated part of the team to aid in the process of continuous improvement (7). For this reason, DE was utilized during the piloting to inform of any changes made to enhance the learning experience and the curriculum’s success. DE guides adaptation through emergent approaches and dynamic realities of program development and implementation, emphasizing an evaluator’s role in shifting methods of dissemination alongside program content changes (8, 9). Since DE utilizes a dynamic approach, it is well suited for complex environments, such as a classroom (10). It fulfills a niche desire in health promotion practice as it continuously creates opportunities to note and address healthy equity concerns in real-time with active participants (8).

### Rationale: development evaluation and *On My Way*

The DE of *On My Way* sought learner-centered feedback in the piloting environment (11). Investing the time for DE at this stage assessed how the program was unfolding, and in turn guided consistent, small-scale adaptations (12). This process sought enhanced communication of the content and a positive influence on students’ attitudes toward communication (13). In the end, both DE and *On My Way* seek the same thing: “learning through knowledge exchange” (14). This dedication to learning is what enables both to be innovative while balancing evaluative processes; furthermore, it creates the desire for long-term learning and capacity-strengthening (14).

## Pedagogical framework

### What a puberty curriculum looks like, and healthy futures of Texas’ role

Much of *On My Way* is linked to the *International Technical Guidance on Sexuality Education* created by the United Nations Education, Scientific, and Cultural Organization (UNESCO). This school-based education guide claims comprehensive sexuality education (CSE) is a pivotal experience for young people preparing to enter puberty. The development of age-appropriate knowledge, attitudes, and skills can be a protective factor (4).

**Figure 1:**
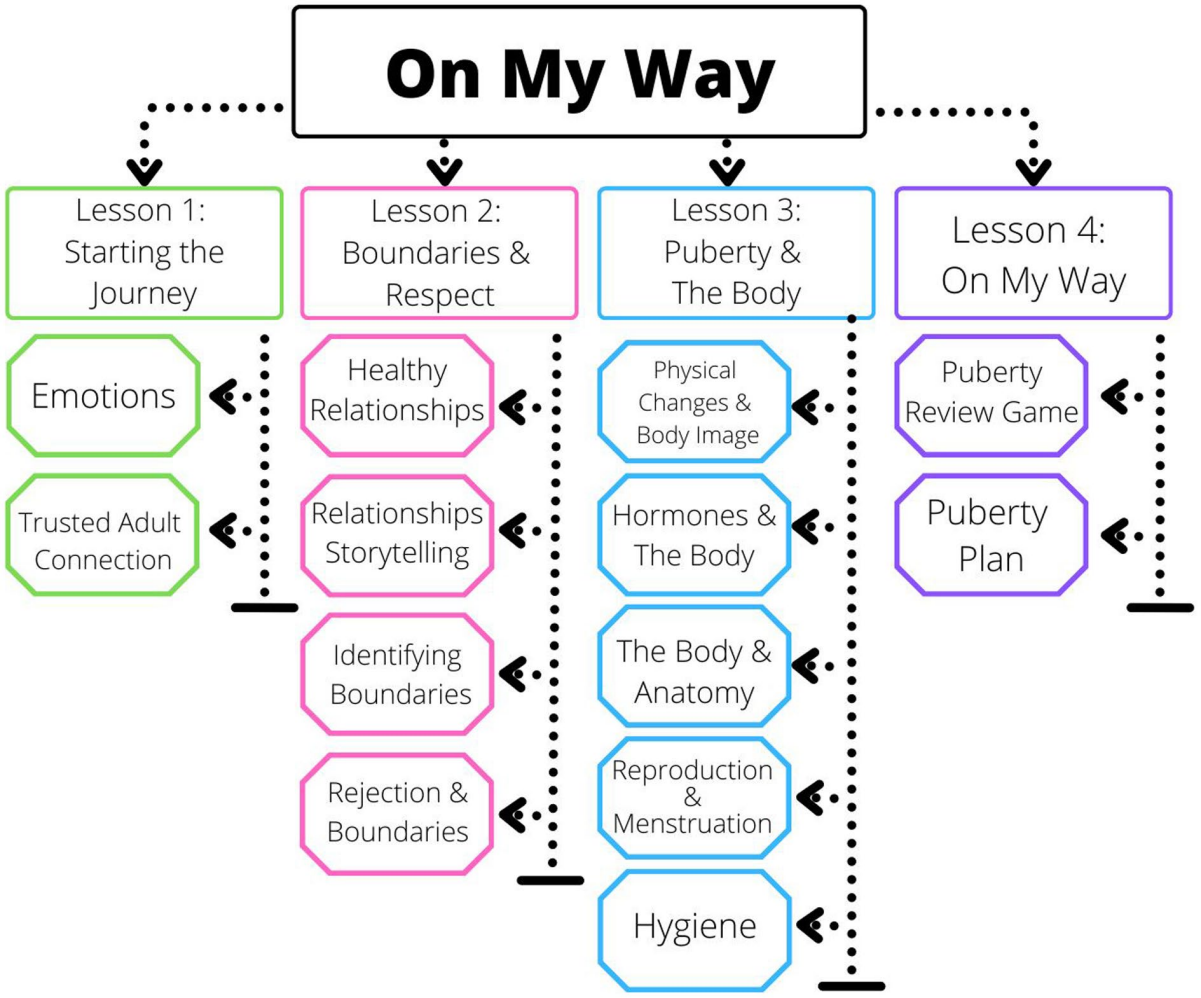
Overview of on my way lessons.

While there is a lack of scientific consensus on how to teach adolescents about puberty, there is much research on why certain information should be included. For *On My Way*, research was divided to account for the physical, emotional, and social changes that occur during puberty. Lessons about social changes covered critical types of communication, relationships, boundaries, and rejection. Some argue that co-ed instruction makes it more difficult to meet specific educational needs; however, it is also noted that gender-separated classrooms can perpetuate the notion that it does not benefit one to learn about the other (5, 15)This notion made it particularly important to combat the stigmatization of bodies and those who look different during the development (i.e., gender, weight, and bullying based on physical changes during development) of the social changes lesson. Emphasizing mood and reaction to the emotional self was important to contextualize the introduction of hormones during puberty. Figuring out how to manage a plethora of new emotions in different contexts is not an easy task, especially for an adolescent, so teaching how to understand these new emotions is imperative. Learning how to understand these new emotions is vital for young people in the early stages of puberty since the part of the brain that regulates those emotions and deep thinking is often the last to develop (Kids (16)). For the physical self and aligned changes (e.g., genitalia and breast development), age-appropriateness of content was important. This research was utilized to create a four-day puberty curriculum, illustrated in Figure 1.

### Theories and approach

This program utilized a research-based approach when developing the curriculum and focused on the action framework of DE during the evaluation process. With a focus on developmental aims, the authors note that a research-based approach recognizes. This approach recognizes the lack of scientific consensus on a topic, but it maintains that existing research is vital as it still holds value and knowledge. When there is a lack of a scientific evaluation to establish findings, one must rely on related findings established through existing research to create a foundation for education purposes (17). To address the gap in research directly related to puberty education in classrooms, the team utilized general research related to adolescent health and development, adapting tools such as UNESCO’s sex education guidelines. The philosophical view of DE is rooted in pragmatism, action, responsiveness, and complexity, which supports contextual observations. With the framework being supportive of learning and decision-making, dialogue between different stakeholders during this process was supported.

The evaluation was conducted through the lens of the Integrated Behavioral Model and noted the pragmatic nature of DE. This methodology considers environmental factors, habits, salience, and knowledge needed to perform what is learned. Keeping these influential factors in mind during implementation and evaluation allows for more contextual observations to be made. For this study, environmental factors such as location and social values (e.g., religion, culture, and political atmosphere) were noted, and knowledge from surveys and observations was collected to learn how outside forces affected the program’s delivery. Acquired knowledge and salience were addressed in the puberty review game and the student survey. Facilitators observed student habits in group discussions and made note of them in the first round of evaluation using a pivot log.

The evaluator completed the following: surveying facilitators, faculty, and students, analyzing survey feedback, researching demographics and general health outcomes of implementation locations, and communicating with the program team on prior research.

## Learning environment

### Implementing *On My Way*

*On My Way* was evaluated at a private school in San Antonio, and at a temporary housing shelter called Haven for Hope. We utilized these locations due to the barriers to implementing pilot health education in public schools in Texas. IRB approval was not needed for this evaluation since there was minimal risk and the scope aligned with category 2 of OHRP Exempt Categories 45 CFR 46.104 - (HRO-312) (18).

Participant ages ranged from 8 to 13. There were 8 participants from the shelter, and 84 were from the private school. A total of 39 participants chose to complete a demographics form. Students filled in their terms for their race/ethnicity. Of those who wrote a response for race/ethnicity, 18 were white, 9 were Hispanic/Latino, 5 were Asian, 2 were Black/African American, and 3 used multiple identifiers. Of those who completed the optional gender line, 18 said “male” or “boy,” and 21 used “female” or girl.” While socioeconomic data were not explicitly collected, one can presume diversity in socioeconomic status between the two pilot sites.

### Evaluation questions

All methods were guided by four evaluation questions that were developed with all potential stakeholders in mind.

1. To what extent is the curriculum adhering to its core operating principles and achieving the right mix of focus areas?
2. How effective and accessible is the curriculum?
3. Are participants satisfied with the curriculum?
4. Will learners be able to apply this curriculum effectively in their lives?

### Data collection

**Figure 2:**
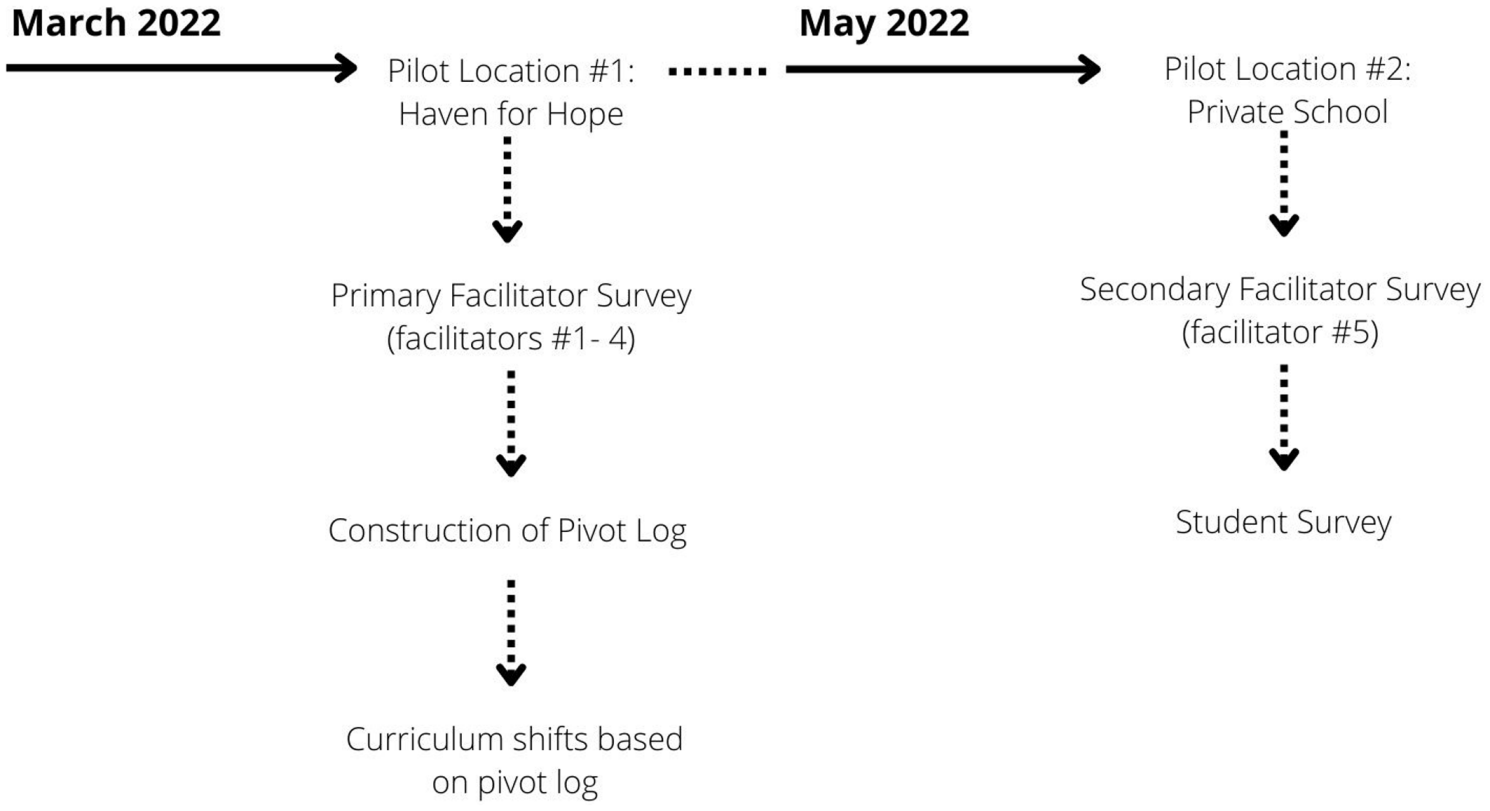
Timeline of pilot events.

Data were collected through facilitator surveys, facilitation notes from implementation, and optional student surveys. Parental consent was gathered prior to implementation. The student survey was distributed at the second pilot site, the private school (see Figure 2), and used purposive sampling, as only a select number of students received the piloted version of *On My Way.* Google Form surveys were circulated among facilitators and faculty who had been present during the sessions. These surveys provided more detailed opinions and reactions to the implementation from those who were able to observe the youth as they participated. Facilitation notes and post-implementation reflections were also collected, detailing observations and challenges. A pivot log was created to note lessons learned from early evaluation tools to advise on changes ahead of the next implementation.

## Results

### Pivot log

Primary facilitator surveys were disseminated after their first time implementing the full curriculum, as indicated in Figure 2. The survey responses were utilized to create a *pivot log* (see Supplementary Appendix), suggesting edits prior to further implementation. Several themes for change were observed and noted by multiple participants.

- Some activities did not have enough time to be effective within the 45 min allotted per session.
- Students expressing discomfort regarding learning about puberty together.
- Reorganization of certain lessons
- Student difficulty grasping concepts or definitions.

### Facilitator survey

Facilitator surveys (see Supplementary Appendix) were completed anonymously by five facilitators at the first time point they were involved. Four of these facilitators were associated with Healthy Futures, and the fifth was the pilot location’s classroom teacher. All had varying levels of experience with the curriculum and facilitation in general. Surveys were created to gauge the facilitator’s comfort level and to gain perspective on the implementation. At the first pilot location, facilitators #1 to #4 were involved and completed their surveys after this implementation. Facilitator #5 began working with the pilot at the second site and completed their survey after. There were 10 survey questions, and all facilitators completed them. Questions were created using a mixed methods approach (see Table 1).

**Table 1:** Representative quotes from qualitative questions from facilitator surveys.

Question	Response
**1. Have you had experience with other puberty curriculum and how does this curriculum compare?**	
	“I have only had experience with the former [Healthy Future’s] curriculum… the first curriculum was very much focused on the science of puberty, [but] this curriculum goes into the who/what/when/where/why/how and feels much more skills and identity focused.”
	“I’ve reviewed curriculum, and I had puberty as a kid. This is much more in depth. Unlike other curriculum like Proctor and Gamble, it seeks to really educate, not just sell deoderant. I also think it’s really well researched and responsive to young people.”
	“This is my second puberty curriculum that I facilitated and it was by far an improvement. The activities felt thoughtful and the topics were relevant and important.”
	“This curriculum utilized different modalities (written, visual, role play) that other curriculums did not. I believe that made it more engaging/interesting/relevant/accessible to student participation and engagement.”
**2. If you felt uncomfortable while facilitating, why?**	
	“I always feel a bit uncomfortable as an observer”
	“It was new, this was my first time facilitating. I always feel bad when I cannot give my students good eye contact because I am reading from a script.”
**3. If you felt disengaged during facilitation, why?**	
	“There were times that the curriculum got lecture-heavy and it felt like I wanted to rush through those parts. The anatomy portion was very difficult to facilitate.”
	“I think some of the activities need work to make them more engaging”
	“Some of the reflections/script felt wordy and/or advanced. Mid-lesson reflections would sometimes be a bit awkward.”
**4. What activities were difficult to facilitate (if any)?**	
	“The boundaries activity proved to be the most difficult. Otherwise, other activities were relatively easy.”
	“The anatomy activity seemed like a challenge”
	“The boundaries and accountability activity did not seem to get through to them. Might need to simplify. I also felt unsure about the Justice and River role play, it was super detailed and I dont think young people this age have the capacity for all of that.”
**5. What activites do you think the students enjoyed the most and enjoyed the least?**	
	“The Green Flags activity seemed to be a class favorite. The Puberty Plan, I would say, fell in the middle with some students feeling very connected to it and others kind of using the time to joke and chat. The Boundaries activity was probably the least favorite and most confusing to the students.”
	“I think they really liked the worksheets. I also believe they really [enjoyed] trusted the adults [activity].”
	“I think they loved the trusted adults activity and the feelings wheel activity. My class was super interested in the menstrual health portion, so I was glad to be able to bring menstrual health products into class so everyone could see and ask questions. In the future, this will happen on the anatomy and reproduction day. The least favorite I think would be the boundaries activity.”
	“Anonymous questions were cited as helpful. Some discomfort around the anatomy sections were also reported.”
	“The [puberty review] game. Because it gave them perspective of everything they actually retained.”

### Student survey

Student surveys (see Supplementary Appendix) were completed after the last day of implementation at the second implementation site. There were a total of 9 questions, 4 of which required an answer, and 20 students completed the survey. A mixed methods approach was utilized again (see Tables 2, 3). Student surveys were not implemented at the first site due to time constraints.

**Table 2:** Quantitative questions and responses from the student survey.

Question	Response category	% of Students (*n* = 20)
**1. Have you talked with anyone about puberty before these sessions (e.g. Teachers, parents, guardians)?**		
	Yes	75%
	No	25%
**2. How comfortable were you during the session on a scale of 1–5?**		
	1 (not at all)	10%
	2	60%
	3	25%
	4	0%
	5 (very)	5%
**3. How much did you enjoy today on a scale of 1–5?**		
	1 (not at all)	40%
	2	20%
	3	25%
	4	15%
	5 (a lot)	0%
**4. How much did you learn from the activities?**		
	Not much	20%
	Some	40%
	A lot	40%
**5. Did you ask a question during the session?**		
	Yes	40%
	No	60%
**6. If you did not ask a question during the session, why?**		
	I asked a question	40%
	Shy	10%
	Did not have a question	15%
	Awkward Conversation	15%
	Did not feel safe asking	0%
	Other/Combination of above responses	20%
**Comparison: student comfort (q2) and learning (Q4)**	1 (not at all)	*N* = 5%, S = 5%
**Using student comfort level scale as the response category**	2	*N* = 15%, S = 25%, *A* = 20%
*N* = “Not Much”	3	*S* = 10%, A = 15%
*S* = “Some”	4	*N*, *S*, *A* = 0%
*A* = “A lot”	5 (a lot)	*A* = 5%

**Table 3:** Qualitative questions and responses from the student survey.

Question	Response category	% of students (*n* = 20)	Quotes
**1. What was your favortie activity and why?**			
	Review game	60%	“The game because it was fun.” “game. Because it wasn’t weird.”
	Mindful eating	20%	“Snack because gummies.” “Eating because the only not uncomfortabe thing.”
	None	15%	“Nun.” “I did not have one.”
	Feelings activity	5%	“Feelings”
**2. What was your least favortie activity and why?**			
	Anatomy slideshow	45%	“Because it was odd.” “slide show. Because pictures.” “the slide show because really awkward.”
	N/A	45%	“I liked all the activites the same.” “I do not know.”
	Q&A sessions	5%	“Questions because its questions.”
	Writing activities	5%	“Because I wanted to hear not write.”
**3. What would you tell a friend who is also going to be learning about puberty?**			
	Support and preparation	45%	“It’s OK to feel the way you feel, it’s normal.” “its weird but everyone goes through it.”
	No advice/Do not know what advice to give	25%	“I do not know.” “nothing.”
	Avoid topic	15%	“do not go!!” “be prepared for things to be sus.”
	Other	15%	“use deoderant.” “all the stuff I learned.” “it would not be as bad if they separated the boys and girls.”

## Discussion

As expected of a DE, many shifts and alterations were made along the way. Student’s perceived desire to learn, session time, and available resources were all major challenges to overcome. *Pivot Logs* allowed for the evaluation team to note any unexpected reactions to the curriculum, before and during the pilot sessions. Later, the *student surveys* brought to light some unexpected findings, even though the curriculum strived to account for context.

### Pivot log

The emoji check-in was found to not be effective in its initial purpose. Students were asked how they felt coming into the lesson, and they responded by holding up a corresponding emoji, but time constraints became an issue. Initially, facilitators were going to debrief the selections made, but shifting the purpose of it to a swift emotional gauge rather than an in-depth check-in was a better balance. A swift emotional gauge allowed students to feel seen while also saving time. However, personalized responses to negative emotions toward the lessons were not able to be mitigated as quickly.

Another issue that facilitators expected was the large number of students who expressed discomfort when boys and girls were not separated for the lessons. The research team was not able to find research that demonstrates students feel safer in single-gender groups, or that learning improves. However, guidance from LGBTQ+ advocacy groups emphasized why youth should be kept together; for example, young people must see it is possible and safe to discuss these issues with students of another gender (5). Based on this, the research team felt the need to emphasize the importance of having all students learn together, rather than in single-gender groups.

Another note was that worksheets will need to be reorganized and edited to allow for a more intentional sequence of topics and activities so that the information being provided is easier for students to build upon over time. Ensuring that the students’ cognitive and emotional abilities were well aligned with the organization of topics. The edited curriculum sequence will be geared toward flexibility, allowing students to engage more in areas where curiosity is piqued, in turn reducing learning fatigue (19). Although gaps in students’ knowledge were acknowledged initially, providing definitions directly in the workbook—rather than providing them as needed aloud—will likely enhance student’s experiences with the curriculum. The addition of definitions to the workbook would limit pauses during the lesson to answer these questions, it will allow students to read and process this information at their own pace, and this will make it easier for students to return to it as needed throughout the sessions.

### Student surveys

Students were quick to note their comfort levels, with 70% of students reporting not feeling comfortable during sessions. While many reported being uncomfortable, a slightly larger majority (75%) have already had prior conversations about puberty. Prior student exposure to this topic has the potential to result in them feeling more comfortable when learning about it later; however, these student surveys revealed that this is not always true.

How students reported enjoying the day and how much they learned can be viewed as contradictory. A total of 40% of students reported that they did not enjoy the day at all, while 80% reported learning something from the lessons. Through evaluation efforts, it can be concluded that even when stepping away from the norm of puberty curricula, it can still fall short of student comfort. Barriers to student comfort in puberty education can be tied to disruptive behavior, the lack of a dedicated classroom or space, insufficient time allotted for lessons, discomfort with relevant terminology, and lack of answering questions proposed by the students (20). While *On My Way* worked to combat these barriers, comfort levels still fell short. We encourage more research on the nature of discomfort in puberty education, what impact it has on the learning experience, and how it can be minimized.

The majority of students (80%) reported learning something during the lesson, yet 55% of the students did not have any advice to give peers. Determining what adolescents find to be important or advice-worthy may be an avenue to look down when breaking down this contradictory data.

### Analysis of evaluation questions

After the second pilot was completed, the extent to which the curriculum adhered to its core operating principles was solidified. While perfect timing is still in progress, the development of clearly defined lessons covering multiple topics has panned out efficiently. *On My Way* remains accessible to the target age group, as students were presented with age-appropriate topics, phrasing, and terminology. Accessibility in this light was guided by the Texas Essential Knowledge and Skills (TEKS) standards and compared to various other puberty education programs with a similar focus (see Supplementary Appendix). Facilitator notes were also added to the curriculum guide to make implementation more accessible to those who have had little experience teaching this subject. Furthermore, there are also plans to have the curriculum printed in Spanish and English, expanding the lens of accessibility across prominent cultures in Texas.

Opposingly, there are some issues regarding effectiveness based on student survey responses. Curriculum shortcomings were predominantly rooted in student comfort level, low confidence in providing advice, and inconsistent enjoyment of activities. Adjustments to the workbooks were made, this includes the addition of definitions and reorganization of order. In turn, lessons will operate more smoothly, creating the opportunity for a more effective implementation.

Initially, the facilitator’s observation of students’ body language indicated mixed results regarding student satisfaction. However, upon receiving the surveys back, observation can be put into more context. *On My Way* is a satisfactory curriculum by way of content in regard to the TEKs, but in terms of student satisfaction, it is low. As *On My Way* currently stands, it is clear that more time needs to be dedicated to building rapport with the students to create an inclusive environment to match the inclusive curriculum.

Mixed results continued into the discussion on whether or not students will be able to apply what they learned. Based on the student survey responses, 45% of students were able to provide peers with good advice on learning about puberty. The issue, however, is that another 40% of students either did not know what advice to give or gave the advice to avoid the topic. This can be linked to the age and emotional intelligence of the students receiving the puberty curriculum. Social perception is a piece of emotional intelligence that is tied closely to the ability for information processing and listening comprehension (21). Further research is needed to determine if certain activities and delivery methods would aid in student comprehension, or if emotional intelligence is the driving force behind an adolescent’s ability to utilize new information quickly.

### DE implications for practice

One of the most prominent lessons learned was that gaining trust in a new community takes time. In order to learn as much about the successes and pitfalls of a program using DE, there is a higher level of interpersonal communication that is required. Creating dedicated time for relationship building was a challenge. From this experience, it would be recommended that relationship building with sites and participants be viewed as a core, first step of the evaluation. There is great strength in connection, and that will show through in evaluation results.

Another lesson revolved around the notion that there is not one way to conduct DE. Being adaptable is essential to DE, but building a strong outline of the evaluation process best suited for the environment proved helpful. Embracing the ever-changing nature is included; however, going into the evaluation process expecting adaptability to guide it can become complicated. For example, while shifting the emoji activity to a swift check-in rather than an in-depth conversation saved time in the session, it took away time to build a deeper connection and trust with the students. Determining how to adapt quickly while maintaining the same outcome can pose a challenge.

A final lesson learned in this evaluation process was that organization and objectivity are key. Organizing thoughts and practices to maintain the same intention throughout changes was a challenge. When shifting implementation practices abruptly, it can be difficult to shift the outlook at the same pace. Ensuring that intentions are organized and adapted while remaining objective in the presented curriculum benefits the reported outcomes of the evaluation process greatly.

## Strengths and constraints

The nature of DE is a clear first strength, as it is adaptable and easily manipulated to fit the context of the puberty curriculum. Its formative approach allowed for user feedback DE sought after, in addition to its ability to fit into a learning environment (23). Another strength is that all facilitator surveys were completed in a timely manner. A final strength is that the evaluator was also a part of the curriculum-building process. Prior knowledge of the information and the program was beneficial when conducting evaluative processes. These strengths allowed the evaluator to focus on the impact of implementation rather than the foundation of the curriculum itself. It also was beneficial when providing feedback to the facilitators on the pivot log.

Alongside these strengths, there were limitations. One was that the implementation sites were in Texas and the evaluator was located in Illinois. The inability to have the evaluator present led to some miscommunication and work delays. Furthermore, all feedback on the sessions was from those participating or those unfamiliar with the curriculum. Having the evaluator present could have led to a greater understanding of what sections students enjoyed more, or how different facilitators approached the curriculum. The sample size of 20 can be viewed as a limitation, given the concern that a small sample size is insufficient for evaluation efforts. However, small sample sizes work in DE since the program should be critiqued before larger-scale development and full implementation (22). Having a smaller sample size allowed facilitators to observe student reactions more easily, as well as try new approaches based on feedback from prior sessions. Another limitation is that we had limited options for pilot sites due to the conservative nature of health education in Texas. The curriculum was designed to be inclusive and comprehensive, but the depth of certain topics, such as gender and contraceptives, was limited to ensure wider acceptance.

Limitations were also found in the structuring of the student survey. The lack of follow-up questions on how to address a student’s reactions in regard to the comfort and relevance of information became a clear issue. This became clear after the second pilot when facilitators noted some student reactions in sessions remained consistent between the pilot sites, even after the program was adjusted to address prior discomfort. If follow-up questions were included, fewer presumptions would have been made in curriculum editing. A final limitation is that student surveys were not completed immediately after the last lesson was taught and lessons were split by the weekend. This gap in the curriculum created by the weekend could have shifted students’ perception of the topic or resulted in the loss of new information.

## Conclusion

DE fosters conversations leading to change and adaptation. Utilizing this evaluation method during the pilot stage of *On My Way* began the work of aligning intention with outcome. A wide research base supporting early implementation gave space to the adaptive nature of DE that sought to improve the *On My Way* curriculum. Including user experience in the evaluation provided some of the most crucial critiques. DE being rooted in teamwork, perspective, and relationships aided greatly in the process of fostering a trusting environment for change. Even though not all participants had the best experiences with *On My Way* during the pilot stages, it is their comments that are going to improve the curriculum and experience for other students. Future research is recommended to build upon the interpersonal roots of this study and seek to determine how it affects student’s knowledge retention.

## Data Availability

The original contributions presented in the study are included in the article/Supplementary material, further inquiries can be directed to the corresponding author/s.
