# Supplementary material for: Developmental evaluation of the healthy futures of Texas’ puberty curriculum: On My Way
Source: Front Public Health. 2024 Sep 25;12:1441326. doi: 10.3389/fpubh.2024.1441326 (PMC11461230; doi:10.3389/fpubh.2024.1441326)
Supplement: Supplementary file 1 [file Data_Sheet_1.DOCX]

**Appendix**

*Integrated Behavioral Model*
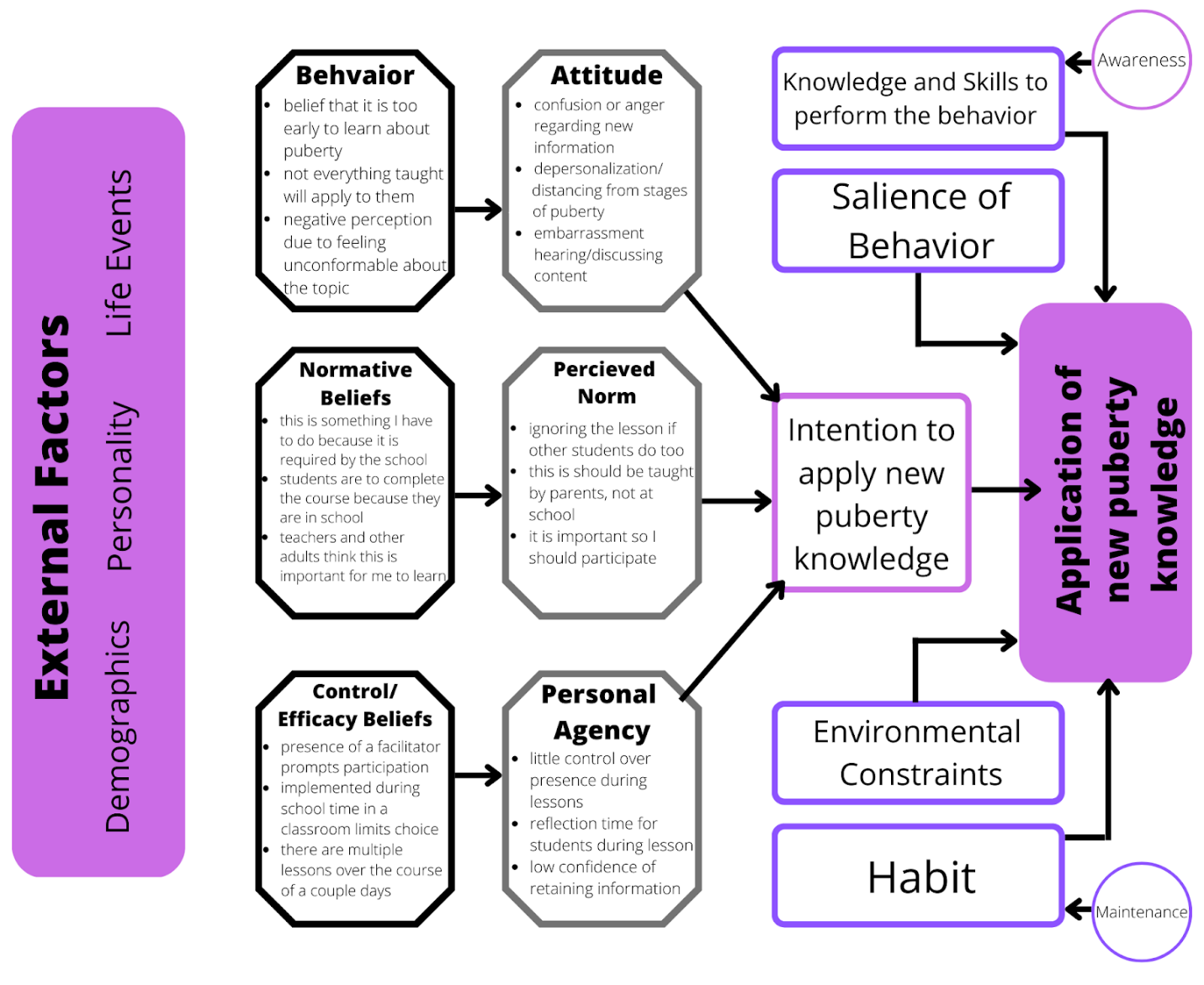
Note. Integrative Behavioral Model for development of *On My Way*. Own work.

*Pivot Log*

| **Date** | **What Changed** | **Reason for the Change** | **Comments** |
| --- | --- | --- | --- |
| 03/15/22 | How puberty is defined and displayed in session | Definition did not meet objective of tying in reproduction and sex | Having a poster with the definition hung on the board behind facilitators, potentially adjust definition: "Puberty is the bodily process of sexual maturity. It is a temporary life stage that includes social, sexual and reproductive development." |
|  | Need to change up Emoji check-in | Held little value, students did not look at cards again after the activity | The way it was mitigated with small discussion was nice - potentially making it a point to have a quick emoji check-in after each mini lesson before moving onto the next one. In a way for facilitators to gauge how the concept was grasped and if more coverage/supplementing is needed |
|  | Group agreements clarity | Desire for poster in background to uphold agreements, and encourage connection of agreements to different lessons | Having a set poster with general agreements, and allowing students to add what they feel is necessary (provide space to write in at bottom of poster or do so on the board next to the poster) and keep up for each session and review at beginning |
|  | Question box | There is not enough time to solicit/collect at the end of each session | Make space for collection throughout lessons- proposal: bring a couple small boxes to each session to sit on each table section/areas of the room to make those who have a question more comfortable putting one in the box as It will be more discrete |
|  | Question Box Cont…. | Use at the end of each session not the beginning of next | Good idea- keeping questions relevant for the days lessons as not to confuse students of topics during the next session |
|  | Desire for glossary | Worries that other groups will not have a good grasp on concepts & words/definitions | To be handed out on the first day- even if students are able to grasp the vocab off the cuff, having something to take home and reference or talk with parents about beings the curriculum to life outside of the classroom |
|  | Reworking the feelings wheel // saving time not handing out papers | Not enough time to pass things out, ended up doing whole group work | Would passing out papers all at the beginning make sense? Having them pre-set up in packets almost for each session to have passed around the room during opening discussion. Like the race to come up with body feelings ! |
|  | Boundaries | Some students are not grasping it | Provide a list of things that can represent boundaries and things that are not boundaries (feelings and personality) - maybe explain that these things are what we use to set boundaries and can be used as "warning signals" of needing to set a boundary/getting close to crossing one    Define boundaries: actions being taken to help yourself feel comfortable and safe in different scenarios? |
|  | Boundaries cont… | Worksheet development - needs more structure and bigger lines | Structured in way of the activities- will aid flow of conversation. Provide definitions on the work sheet? |
|  | Worksheet reorganization | Color wheel needed in black & white, enlarging images (Venn diagram), design own color wheel? | All easy fixes. What would designing own color wheel accomplish? Does the one currently being used not meet every need? What would be different about it? |
|  | Addressing different gender expression in lessons | Desire for all students to benefit from each lesson and feel like they apply/differing identity can be taken into consideration | Don’t add "or whatever gender makes the most sense for you" make that the only instruction. "Imagine you in this scenario as whatever gender makes the most sense in this moment" |
|  | Need to loop back to previously instructed concepts | Needed to ensure understanding since concepts build off one another | "Remember how we identified a trusted adult? How can we create boundaries to ensure that we maintain green flags and keep out red flags?" |
|  | Prep students for Why they are learning this | Ensure that the way in which the curriculum is conducted is beneficial to them in the long run | Make it a brief section alongside the definition on the first day, or simply just remind them and connect to each lesson "keep in mind why we are talking about [boundaries], sometimes throughout life we are tested and need to remind ourselves to be firm in what is right for us…." |
|  | Hygiene lesson was too short & low engagement | Need to ensure the time is used to it full ability, if students are not grasping or participating then change is needed | Make it more interactive If low engagement- ex: when defining hygiene maybe have a list of words surrounding the topic with some a little disconnected and have students "cross out" the ones that do not apply? -- will not only use up time but also create more engagement/active thinking |
| 03/28/22 | Day 2: too lecture heavy/exhausting    need to provide resources, create time for group discussion, desire for space to practice rejection skills, and address how to end a relationship | To make the session more engaging and to ensure that students are learning to the best of their abilities. The addition of resources makes this portion more accessible. | Would the creation of a resources page in the booklet make sense?  Working group response into lecturing may make it feel more engaging and tackle "checking-out" -- potentially using the emoji's as a quick check in throughout? |
|  | Desire to give more time to practicing healthy coping skills for negative emotions | Important for adolescent growth and understanding of self | Consider replacing an activity? Would exchanging this idea and boundaries make sense? (in terms of time spent on activity) |
|  | Need to determine the inclusion of "romance" and "romantic relationships" in these activities | Mixed comments on its inclusion. While important to touch on all types of relationships, is it beneficial if the students are confused and embarrassed by it? | Potentially just mentioning romantic relationships and an example from the facilitator, but all other expectations being about other relationships from the students? |
|  | Relationships and boundaries activity ran out of time for role play portion, and the relationship was a distraction | In order for students to get the most out of this section, changes are needed  Such as: focus on 1 of the two characters? Already have boundaries and responses for one of them.  Processing questions to be more detailed/leading | Potentially focus on one of the two activities (either the role play or the creation of characters boundaries), reliance on the 4 pillars does not feel to be an issue    For processing questions, make it more of a reflective activity since they are uncomfortable questions. |
|  | Emoji check-in VS feelings wheel | Use of one or the other seems to make the most sense as students were very receptive to the wheel and the use of both would be redundant | The use of either as a check-in throughout the session would be a nice tool, especially when engagement is low, or students look confused |
|  | Rounding out the "boundaries" section | Desire to have more active interest in the section, as well as ensure that definitions are comprehensive based on age | The suggestion of opening the section with a conversation on healthy relationships is a good idea to bridge the potential knowledge gap. Defining types of relationships (having students yell out ones they know…) and providing    For river and justice - potentially provide the students with their boundaries and then have then role play the given boundaries OR have then create the boundaries and leave it at that with reflection questions |
|  | Put "say what" ice breaker explicitly into the curriculum | Aids in tying together portions of the session and concepts learned, also beneficial for all proceeding use of this curriculum | Provide examples of important concepts to touch on in this activity on the facilitator work page for the activity |
|  | Rearrangement of anatomy content | Needed to flow in terms of how the anatomy works- aids in easier understanding    Simplifying the definition of sex/fertilization    Separating learning objectives to ensure delivery is sufficient | Anatomical flow may not always make the most sense when disseminating new information (as mentioned in the notes), but using this as a basis for order will be helpful in more ways than not    The simplified version of the definition makes sense and is easier to grasp    Ensuring that each learning objective has an activity/page/lecture/moment in the session |
| 03/29/22 | Editing the approach to body image | Move to end of lesson and open up next session with discussion    Tie into the group agreements    Emphasize how maintaining a healthy body image is important during puberty | Emphasizing the cycle of self-deprecation/poor self-esteem related to body image effecting how you interact with others, but these physical changes being a result of puberty --- looping back and connecting topics |
|  | Adaptation of activity for hygiene | Desire to rearrange sequence of information  Addition of an activity to this section for engagement  Inclusion of hygiene kits to handout | The addition of a quick and easy activity I think is a great fit for here- I like the sticky note suggestion, but if time is a concern during this session the "twin" worksheet will get the same message across while saving the time of students traveling up to the board |
|  | Menstruation topic issues | Students did not understand why menstruation was relevant to "boys" | Perhaps starting with the directive questions of "how can you support someone who is menstruating? Why is it important to understand menstruation even if you do not go through it?" -- providing a response or two at the beginning will also allow for students to brainstorm similar reasonings to share out |
|  | Day 4 Emoji Check in | There is a lack of follow up on student’s emotions from this activity | Not sure if follow up on this check in is more important than its inclusion - setting the tone by asking their feelings entering this session I believe is important for gauging engagement and participation … even if there is no follow up on negative emotions, potentially using the emoji check-in or feelings wheel throughout the session as a moment to orient energy/time/focus could be beneficial |
|  | Shifting gender identity section | Moving it to Body Image discussion | I think this is a good option and would make for easier connection without direct ties to puberty -- it would also elongate the body image section, potentially mitigating how brief it is currently |
|  | Puberty Survival Skills issues | Use of "survival" was very distracting    Ineffective activity | Elimination of the word "survival" would not change the meaning of the session, also "puberty toolbox" would also be sufficient    Restructuring the activity seems to be the way to go -- potentially a word bank added into the workbook next to the box would help (including words/phrases that are incorrect) and then discussion on where each work was placed? (me, others, relationships) |
|  | Edits to Puberty Plan | Need more facilitator contribution of definitions and reading of prompts    Addition of school resource support and outside resources | Include definitions in workbook (?) and begin by discussing what is a community and how one contributes to it - similar take for other confusing prompts    Addition of resources could be a part of the workbook as well or given as a supplemental handout based on implementation location if specific school based |

*Facilitator Survey*

1. What is your experience with puberty curriculum and how does On My Way compare?
   1. Yes
   2. No
      1. If so, how does it compare to others you have worked with?

_______________________________________________________

1. How comfortable were you while teaching/being in the room during On My Way sessions?
   1. Yes
   2. No
   3. Sometimes
      1. If so, when and why? ______________________________________

________________________________________________________

1. How often could you visually see how comfortable the students were (e.g. body language, facial expressions..)?
   1. Always
   2. Never
   3. Sometimes

1. How engaged were the students during the sessions?
   1. Circle how engaged you think students were:
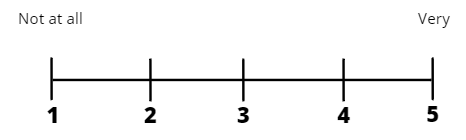


1. As a facilitator did you ever feel disengaged?
   1. Yes
   2. No
   3. Sometimes
      1. If so, why? _____________________________________________

1. Was the session on time?
   1. Always
   2. Sometimes
   3. Never

1. How often did students ask questions?
   1. Often
   2. Sometimes
   3. Never

1. How smoothly did all the activities run (not taking timing into account)? What activities did you have trouble facilitating?
   1. Circle how smoothly today ran:
   2. When was there issues facilitating? ______________________________

1. How effective did you find the activities?
   1. Circle how effective you found the activities:
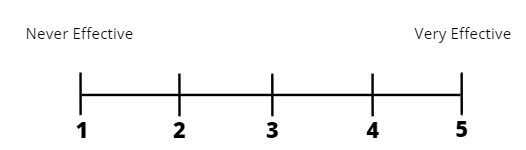


1. What activities do you think the students enjoyed the most and enjoyed the least?
   1. Most enjoyed? _______________________________________________
   2. Least enjoyed?______________________________________________

*Student Survey*

1. Have you talked with anyone about puberty before these sessions (e.g. teachers, parents, guardians)?
   1. Yes
   2. No

1. How comfortable were you during the session?
   1. Circle how comfortable you were:
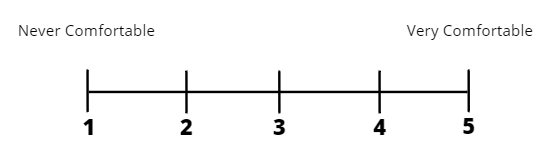

2. How much did you enjoy today?
   1. Circle how much you enjoyed today:
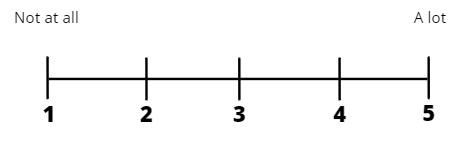


1. How much did you learn from the activities?
   1. Not much
   2. Some
   3. A lot

1. What was your favorite activity and why?
   1. Favorite activity? ________________________________________
   2. Why? _________________________________________________

1. What was your least favorite activity and why?
   1. Least favorite activity? ___________________________________
   2. Why? _________________________________________________

1. What would you tell a friend who is also going to learn about puberty?
   1. ______________________________________________________

1. Did you ask a question during the session? If not, why?
   1. Yes
   2. No
   3. If not, why?
      1. Shy
      2. Did not have a question
      3. Awkward conversation
      4. Did not feel safe asking
      5. Other: _____________________________

*TEKs Alignment*

**115.6. Health Education, Grade 4**

Grade 4:

- TEKS 4, G

Health behaviors. The student understands and engages in behaviors that reduce health risks throughout the life span. The student is expected to:

- G) identify types of abuse such as physical, emotional, and sexual and know ways to seek help from a parent and/or trusted adult.
- Covered in Activities 1.3, 2.2, and 2.3
- TEKS 8, A

Personal/interpersonal skills. The student understands how relationships can positively and negatively influence individual and community health. The student is expected to:

- A) explain the influence of peer pressure on an individual's social and emotional health
- Covered in Activity 2.4
- TEKS 9, A & C & E & H

Personal/interpersonal skills. The student uses social skills for building and maintaining healthy relationships throughout the life span. The student is expected to:

- A) describe the qualities of a good friend
- Covered in Activity 2.2
- C) explain the importance of refusal skills and why the influence of negative peer pressure and the media should be resisted;
- Covered in Activities 2.3 and 2.4
- E) identify critical issues that should be discussed with parents/trusted adults such as puberty, harassment, and emotions;
- Covered in Activity 1.3 and Lesson 3
- H) demonstrate refusal skills.
- Covered in Activity 2.3
- TEKS 10, C

Personal/interpersonal skills. The student explains healthy ways to communicate consideration and respect for self, family, friends, and others. The student is expected to:

- describe strategies for self-control and the importance of dealing with emotions appropriately and how they affect thoughts and behaviors.
- Covered in Activities 1.2 and 2.4
- TEKS 11, A & C

Personal/interpersonal skills. The student demonstrates critical-thinking,

decision-making, goal-setting, and problem-solving skills for making health-promoting

decisions. The student is expected to:

- A) explain the importance of seeking guidance from parents and other trusted adults in making healthy decisions and solving problems;
- Covered in Activity 1.3
- C) describe the importance of parental guidance and other trusted adults in goal setting
- Covered in Activity 1.3

**115.7. Health Education, Grade 5**

Grade 5:

- TEKS 1, F:

Health information. The student knows ways to enhance and maintain personal health throughout the lifespan. The student is expected to:

- F) analyze the components of a personal health maintenance plan for individuals and families such as stress management and personal safety
- Covered in Activities 4.2 & 4.3
- TEKS 2, B:

The student recognizes the basic structures and functions of the human body and how they relate to personal health throughout the lifespan. The student is expected to:

- B) identify and describe changes in male and female anatomy that occur during puberty
- Covered in Lesson 3
- TEKS 5, H:

Health behaviors. The student comprehends behaviors that reduce health risks throughout the lifespan. The student is expected to:

- describe the value of seeking advice from parents and educational personnel about unsafe behaviors
- Covered in Activity 1.3
- TEKS 6, A, B, D:

Influencing factors. The student understands how relationships influence individual and family health including the skills necessary for building and maintaining relationships. The student is expected to

- A) distinguish between healthy and harmful influences of friends and others
- Covered in Activities 1.3, 2.2, & 2.3
- B) describe the characteristics of healthy and unhealthy friendships
- Covered in Activities 2.2 and 2.3
- D) analyze respectful ways to communicate with family, adults, and peers
- Covered in 1.2, 2.3, and 2.4
- TEKS 8, A:

Influencing factors. The student knows how various factors influence individual, family, and community health throughout the lifespan. The student is expected to:

- A) explain the importance of communication skills as a major influence on the social and emotional health of the individual and family
- Covered in Activities 1.2 and 2.4
- TEKS 9, A & B:

Personal/interpersonal skills. The student demonstrates critical-thinking, decision-making, goal-setting and problem-solving skills for making healthy decisions. The student is expected to:

- A) describe health-related situations that require parent/adult assistance such as a discussion of the health-related consequences of high-risk health behaviors or going to a doctor;
- Covered in Activity 1.3 and throughout Lesson 3
- B) assess the role of assertiveness, refusal skills, and peer pressure on decision making and problem solving
- Covered in Activities 2.2, 2.3, and 2.4

**115.26. Grade 6, Adopted 2020**

Grade 6:

- TEKS 2, A & C

Physical health and hygiene--personal health and hygiene. The student understands health literacy, preventative health behaviors, and how to access and evaluate health care information to make informed decisions. The student is expected to:

- A) compare immediate and long-term effects of personal health care choices such as personal and dental hygiene;
- Covered in Activity 3.6
- C) describe ways to demonstrate decision-making skills based on health information;
- Covered in Activities 4.2 and 4.3

- TEKS 3, A & B

Mental health and wellness--social and emotional health. The student identifies and applies strategies to develop socio-emotional health, self-regulation, and healthy relationships. The student is expected to:

- A) demonstrate healthy methods for communicating emotions in a variety of scenarios;
- Covered in Activities 1.2 and 2.2
- assess and demonstrate healthy ways of responding to conflict;
- Covered in Activity 2.4
- TEKS 5, A

Mental health and wellness--risk and protective factors. The student recognizes the influence of various factors on mental health and wellness. The student is expected to:

- A)identify and discuss how adolescent brain development influences emotions, decision making, and logic; and
- Covered in Activities 1.2 & 3.2
- TEKS 20, A & B & C & D & E

Reproductive and sexual health--healthy relationships. The student understands the characteristics of healthy romantic relationships. The student is expected to:

- A) define and distinguish between friendship, infatuation, dating/romantic relationships, and marriage;
- Covered in Activity 2.2
- B) describe how friendships provide a foundation for healthy dating/romantic relationships;
- Covered in Activities 2.2 and 2.3
- C) list healthy ways to express friendship, affection, and love;
- Covered in Activities 1.2, 2.2, and 2.3
- D) describe characteristics of healthy dating/romantic relationships and marriage, including sharing, kindness, honesty, respect, trust, patience, communication, and compatibility;
- Covered in Activities 2.2 and 2.3
- E) explain that each person in a dating/romantic relationship should be treated with dignity and respect; and
- Covered in Activities 2.2 and 2.3
- TEK 21, C & E & G

Reproductive and sexual health--personal safety, limits, and boundaries. The student understands how to set and respect personal boundaries to reduce the risk of sexual harassment, sexual abuse, and sexual assault. The student is expected to:

- C) list the characteristics of unhealthy or harmful relationships, including anger, controlling behavior, jealousy, manipulation, and isolation
- Covered in Activity 2.2
- E) explain how a healthy sense of self and making and respecting decisions about safe boundaries and limits promote healthy dating/romantic relationships;
- Covered in Activities 2.3 and 2.4
- G) explain the importance of clearly communicating and respecting personal boundaries and using refusal skills related to physical intimacy such as holding hands, hugging, and kissing.
- Covered in Activities 2.3 and 2.4
- TEKS 22, A & B & C & D

Reproductive and sexual health--anatomy, puberty, reproduction, and pregnancy. The student analyzes adolescent development, the process of fertilization, and healthy fetal development. The student is expected to:

- A) describe changes in male and female anatomy and physiology during puberty and how rates and patterns of development can vary between individuals;
- Covered in Activities 3.1 and 3.3
- B) describe the process, characteristics, and variations of the menstrual cycle;
- Covered in Activity 3.5
- C) analyze the role of hormones related to growth and development and personal health;
- Covered in Activity 3.2
- D) describe the cellular process of fertilization in human reproduction;
- Covered in Activity 3.4
